# 

**Published:** 1884-07

**Authors:** 


### Contents—July.

ORIGINAL COMMUNICATIONS:

Fermentation in the Human Mouth. W. D. Miller .......... 339
Proper Medical Education .......... 348
Microscopical Studies upon the Absorption of the Roots of Temporary Teeth. Frank Abbott .......... 349
Dental Education. Rejoinder. Faculty Baltimore College Dental Surgery .......... 358
Pivot Crown. F. E. Howard .......... 368

REPORTS OF SOCIETY MEETINGS:

Southern Dental Association .......... 369
Illinois State Dental Society .......... 374
American Medical Association .......... 380

EDITORIAL:

To Contributors .......... 391

The Half Year .......... 392

Personal—Our Colleague .......... 392

An Important Decision .......... 393

CURRENT NEWS AND OPINION:

The Dinner to Dr. Miller .......... 394
Supreme Court of Illinois .......... 395
Meeting of Delegates .......... 398
New Jersey State Dental Society .......... 399
Pennsylvania State Dental Society .......... 399
Dentists' Benevolent Association .......... 400
National Association of Dental Examiners .......... 400
Society Meetings for July, 1884 .......... 401
American Dental Association .......... 401
Changes Produced in the Teeth by Syphilis .......... 401
Askings and Answers .......... 402

